# Massachusetts Medical College

**Published:** 1846-12

**Authors:** 


*Massachusetts Medical College.*—The lecture term of the Mass. Med. College commenced on the first of Nov., in the newly erected edifice. Hon. Edward Everett, President of Harvard University, delivered an address on the occasion, and was followed by Dr. Hayward, Prof. of Clinical Surgery.

